# Long-Term Prognostic Value of Post-Revascularization Fractional Flow Reserve and Skin Perfusion Toe Pressure in Patients with Chronic Limb-Threatening Ischemia

**DOI:** 10.3390/medicina61091719

**Published:** 2025-09-22

**Authors:** Alexandru Achim, Jeffrey Shi Kai Chan, Szilárd Róna, Ádám Csavajda, Mónika Deák, Gábor G. Tóth, Róbert Bellavics, Attila Nemes, Zoltán Ruzsa

**Affiliations:** 1 Department of Internal Medicine, Invasive Cardiology Division, University of Szeged, 6725 Szeged, Hungary; 2 Department of Cardiology, “Niculae Stancioiu” Heart Institute, University of Medicine and Pharmacy “Iuliu Hatieganu”, 400012 Cluj-Napoca, Romania; 3 Cardiovascular Analytics Group, UK-China Collaboration, Hong Kong, China; 4 Bács-Kiskun County Hospital, 6000 Kecskemét, Hungary; 5 Department of Cardiology, University Heart Center Graz, 8036 Graz, Austria

**Keywords:** critical limb ischemia, chronic limb-threatening ischemia, peripheral artery disease, fractional flow reserve, skin perfusion toe pressure, laser Doppler

## Abstract

*Background and Objectives*: The impact of peripheral below-the-knee (BTK) fractional flow reserve (FFR) on long-term clinical outcomes remains unknown. *Materials and Methods*: We enrolled 40 patients with severe BTK lesions (Rutherford 4–6). FFR (using 40 mg papaverin) and skin perfusion toe pressure (SPTP) by laser Doppler were measured during the index procedure. The primary outcomes were major adverse limb events (MALEs) (defined as reintervention on the index arterial segment or amputation of the index limb) and death during follow-up. *Results*: The median follow-up was 7 [IQR 4–8] years. After the index procedure, FFR increased significantly (*p* < 0.001) and post-revascularization SPTP was significantly higher in the FFR ≥ 0.80 group (*p* = 0.022). Multivariable regressions showed no association between change in FFR (absolute or percentage) and the risk of death (*p* = 0.39, *p* = 0.28) or MALEs (*p* = 0.83, *p* = 0.29), but both pre- and post-revascularization FFR values could predict MALEs at follow-up (*p* = 0.018, *p* = 0.012). Lower SPTP was also associated with the risk of MALEs (*p* = 0.027). SPTP > 97.8 mmHg was 100% specific for FFR ≥ 0.80. *Conclusions*: While there is no association between change in FFR and the risk of death or MALEs, lower FFR values either before or after revascularization were associated with higher long-term risk of MALEs. Moreover, a lower SPTP was associated with a higher risk of MALEs. Aiming for approximately 100 mmHg in SPTP represents a non-invasive surrogate of FFR ≥ 0.80. Larger studies are needed to validate the impact of post-revascularization FFR-SPTP-adjacent values on clinical outcomes.

## 1. Introduction

Fractional flow reserve (FFR) is an invasive physiological index that quantifies the hemodynamic significance of a vascular stenosis, calculated as the ratio of mean distal to proximal pressure across a lesion during maximal hyperemia. Although well-established in guiding coronary revascularization [1,2], FFR is underexplored in peripheral percutaneous transluminal angioplasty (PTA). Revascularization in peripheral PTA is guided by angiography only, possibly using indirect functional indices such as Pd/Pa (mean distal pressure/mean proximal aortic pressure) ratio catheter-derived pressure gradient or direct transpedal pressure (which requires direct transpedal access) [3,4]. Nonetheless, the use of pressure guidewires in peripheral revascularization may be particularly valuable in below-the-knee (BTK) lesions, as advancing a catheter to measure the conventional translesional pressure (based on the systolic gradient) is technically challenging without inducing wedge pressure. Moreover, the blood flow reserve at the BTK level—where three main arteries are often well-collateralized—remains poorly characterized. Previously we demonstrated that peripheral FFR measurement is feasible and correlates well with standard morphological and indirect functional parameters (percentage area and diameter stenosis percentage change in transcutaneous oximetry and skin perfusion toe pressure [SPTP]) [5]. In this hypothesis-generating pilot study we aimed to investigate the long-term prognostic value of FFR and SPTP at index procedure.

## 2. Methods

This prospective single-center cohort study was approved by an institutional review board and was conducted in accordance with the Declaration of Helsinki. All underlying data are available on reasonable request to the corresponding author. We included consecutive patients with chronic limb-threatening ischemia (CLTI) (Rutherford 4–6) and angiographically proven arterial stenosis of the distal lower limb (i.e., BTK lesions with ≥70% stenosis by diameter) AND no other severe inflow disease (e.g., femoral/iliac) who underwent peripheral PTA (“revascularization”) in 2014–2016. Patients with complete BTK chronic total occlusions (of all three major BTK arteries), severe diabetic foot syndrome and necrotic distal lower limb were excluded from this study (non-viable tissue distal to the stenosis that may interfere with the physiological assessment). If chronic total occlusions were present, FFR was determined in the 1–2 artery, which was still permeable, but with stenosis. The primary exposure of interest was peripheral FFR, which was measured using a 0.014″ pressure wire (Certus, St. Jude Medical, St. Paul, MN, USA) before and after PTA (injecting 40 mg papaverine) and compared with laser Doppler STPT (PeriFlux System 500, Järfälla, Sweden). The secondary exposure of interest was postprocedural SPTP. FFR was analyzed as both a continuous and binary variable.

As there is no consensus on the cut-off value for peripheral FFR, we used a cut-off of 0.8 for binarization, as this cut-off is used in coronary angiography. FFR was defined as the lowest Pd/Pa ratio following administration of papaverine, averaged over five cardiac cycles. Tracings exhibiting significant artifacts, dampened waveforms, or non-correctable sensor drift were excluded from the analysis. Our Institutional Review Committee ap proved the study (Hungarian National Ethical Agreement IV/8048-1/2021/EKU), and all patients provided written informed consent prior to study inclusion. This study complied with the 1975 Declaration of Helsinki.

All patients were followed up at least yearly from the day of revascularization until December 2023 or death, whichever occurred earlier. The outcomes of interest were major adverse limb events (MALEs; a composite of reintervention on the index arterial segment and amputation of the index limb, whichever occurred earlier) and mortality during follow-up. All outcomes were ascertained by a trained clinician reviewing medical records.

Continuous variables were summarized as medians and interquartile ranges (IQRs), while categorical variables were summarized as counts and proportions. Pre- and post-revascularization FFR measurements were compared using the Wilcoxon signed rank test, and SPTP was compared between groups using the Mann–Whitney U test. The association between exposures (pre- and post-revascularization FFR, absolute and percentage changes in FFR, and postprocedural SPTP) and the cumulative incidence of MALEs was assessed using multivariable Fine–Gray competing regression with mortality as the competing event. Sub-hazard ratios (SHRs) and 95% confidence intervals (CIs) were used as summary statistics. The association between exposures and the risk of mortality was assessed using multivariable Cox regression, with hazard ratios (HRs) and 95% CIs used as summary statistics. The cumulative freedom from MALEs was visualized using Kaplan–Meier curves. All regressions were adjusted for pre-specified covariates, including sex, age, body mass index, smoking status, hypertension, dyslipidemia, renal impairment, diabetes, coronary artery disease, chronic obstructive pulmonary disease, atrial fibrillation, artery revascularization, and Rutherford classification, with the covariance estimator accounting for clustering by the year of procedure. All covariates were ascertained by a trained clinician reviewing medical records. All *p*-values were two-sided, with *p* < 0.05 considered statistically significant. All analyses were performed using Stata v16.1 (StataCorp LLC, College Station, TX, USA).

## 3. Results

**Figure 1:**
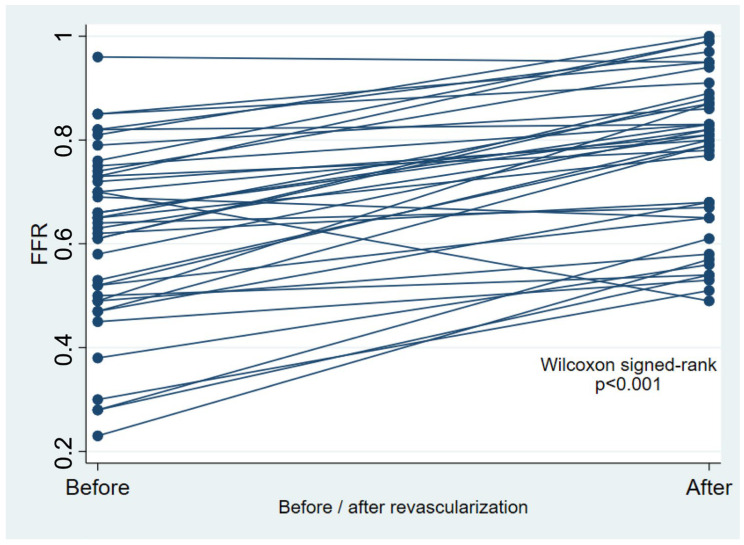
Paired line plot of pre- and post-revascularization functional flow reserve (FFR).

**Figure 2:**
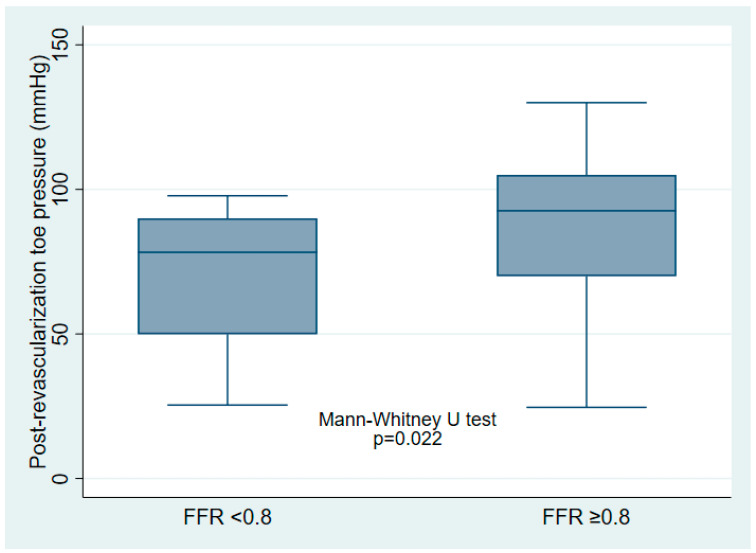
Box plots of post-revascularization skin perfusion toe pressure, stratifying by post-revascularization functional flow reserve (FFR) ≥0.80 or <0.80.

Altogether, 40 patients were included, whose clinical and procedural characteristics are summarized in Table 1. Peripheral FFR improved significantly after revascularization (*p* < 0.001, Table 1 and Figure 1), with a median post-revascularization SPTP of 86 (IQR 60–96) mmHg. Post-revascularization SPTP was significantly higher in the post-revascularization FFR ≥ 0.80 group (93 [IQR 70–105] mmHg vs. 78 [IQR 50–90] mmHg, *p* = 0.022) (Figure 2).

**Figure 3:**
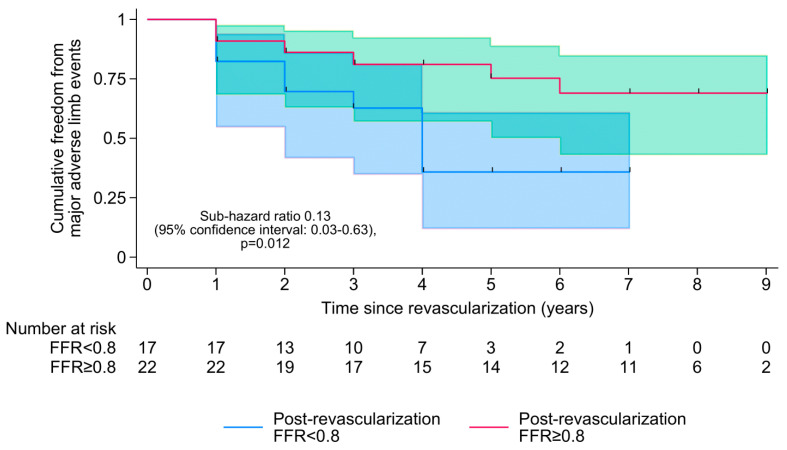
Kaplan–Meier curves showing the cumulative freedom from major adverse limb events over the study period, stratifying by post-revascularization functional flow reserve (FFR) ≥ 0.80 or <0.80.

During a median follow-up of 7 [IQR 4–8] years, 23 (57.5%) patients died and 15 (37.5%) experienced MALEs. There was no loss to follow-up. Out of the 17 patients still alive at the end of the study, 12 (70.6%) had a post-revascularization FFR of ≥0.80, and a median SPTP of 90 [IQR 68–97] mmHg. The results of the multivariable survival analysis are summarized in Table 2. Both pre-revascularization FFR (SHR per 0.1-increment in FFR: 0.65 [95% CI 0.46–0.93], *p* = 0.018) and post-revascularization FFR (SHR per 0.1-increment in FFR: 0.40 [0.19–0.82], *p* = 0.012) were associated with MALEs but not mortality, with a post-revascularization FFR of ≥0.80 being associated with an estimated 87% reduction in the cumulative incidence of MALEs (SHR 0.13 [0.03–0.63], *p* = 0.012; Figure 3). A lower post-revascularization SPTP was also associated with a higher cumulative incidence of MALEs (SHR per 10 mmHg-increment in SPTP: 0.83 [0.71–0.98], *p* = 0.027), with a post-revascularization SPTP of ≥100 mmHg being 100% specific for a post-revascularization FFR of ≥0.80. However, changes in FFR (absolute or percentage) were not prognostic of either MALEs or mortality.

## 4. Discussion

To the best of our knowledge, studies exploring FFR in BTK lesions are scarce [5], and none have explored the associations of peripheral FFR with clinical outcomes. We could demonstrate through our findings that, although the transformation of FFR during PTA could not be associated with overall survival or MALEs during long-term follow-up (7 years), the actual pre- AND post-revascularization FFR values were associated with MALEs (but not mortality), with a post-revascularization FFR of ≥0.80 being associated with an almost 90% reduction in the cumulative incidence of MALEs. This hypothesis-generating pilot study thus represents the first evidence that peripheral FFR in BTK lesions has important long-term prognostic value, and it is an important tool to quantify the supply–demand mismatch both before and after revascularization.

Clinically, peripheral FFR is sometimes used in the ilio-femoral system, but only for assessing the hemodynamic relevance of selected lesions and not for guiding revascularization [6]. Banerjee et al. were the first to correlate peripheral FFR with ankle-brachial index in patients with isolated superficial femoral artery disease with no inflow or outflow lesions [7]. Lotfi et al. demonstrated the correlation of peripheral FFR with peak systolic velocity and the risk of restenosis at a 1-year follow-up [8]. Both studies contained limited observational data (with a sample size of under 20 patients). In a more recent case series of four patients with CLTI and BTK lesions, only the two patients with a post-PTA FFR of ≥0.90 had experienced quick wound healing [9].

The FFR cut-off of 0.80 was chosen as per coronary data, as well as because the Pd/Pa at rest remains fairly constant across the spectrum of stenoses below 80% luminal narrowing [10] in the periphery; however, peripheral FFR thresholds may be lower, as recent data indicate that CLTI tends to occur at FFR values below 0.60 [11]. These data, however, are limited to iliac and femoral lesions [11]. Murata et al., in a cohort of 22 patients with iliofemoral lesions, reported 0.85 as the optimal FFR cut-off for identifying hemodynamically significant disease [12]. Ikeoka et al. demonstrated that a FFR value below 0.88 could predict the presence of ≥75% area stenosis as measured by intravascular ultrasound (IVUS) [13]. A threshold for peripheral FFR may exist to guide the revascularization of moderate-severity stenoses, analogous to the FFR < 0.80 used in coronary vessels. However, peripheral FFR is likely to be, first and foremost, vessel-specific, and may be further influenced by the extent of collateralization and the viability of the downstream tissue [14].

In a similar BTK study (without hyperemic indexes), higher post-procedural resting Pd/Pa values were associated with improved wound healing and clinical outcomes, although the differences did not reach statistical significance [15]. These findings suggest a complementary role for FFR in revealing the hemodynamic significance of certain lesions and in guiding the overall results of PTA.

Pending further studies and more robust evidence, pressure wire measurements in the peripheral circulation may offer numerous potential clinical applications: (1) to assess the stenosis severity of indeterminate lesions and therefore guide revascularization procedures; (2) to assess the immediate efficacy of the revascularization procedure and predict long-term outcomes; (3) to assess the individual contribution of each stenosis in segments that contain long, diffuse disease; and (4) to address the existing disparity in assessing the adequacy and extent of perfusion both before and after revascularization for wound healing [16,17,18,19].

In addition to the above findings, we demonstrated that post-revascularization SPTP may be an alternative prognosticator of MALEs and a specific surrogate for FFR. Overall, our results may pave the way for further studies on broadening the use of FFR to peripheral revascularization, as well as non-invasive and more accessible prognosticators in peripheral revascularization.

This study has several limitations. The single-center nature and small sample size precluded definite conclusions from being drawn and meant that our findings should be seen as hypothesis-generating. The observational nature also meant that the study was predisposed to residual confounding, despite having accounted for a large number of confounders by means of multivariable adjustments. These may include other factors that may influence peripheral FFR, such as the number and patency of collaterals, damages to the microvasculature and tissue diseases (infection, inflammation, and diabetic microangiopathy), and a rheological state (a higher wedge pressure). These factors’ effects on peripheral FFR are underexplored and warrant greater focus from the scientific community. The presence of ipsilateral CTOs contributing to the CLTI state was present in 40% of our patients, explaining the Rutherford 5–6 classifications and the multilevel pathology. This also highlights that more severe and diffuse disease (triple BTK CTOs, severe diabetic foot) lesions were excluded from this study. Lastly, post-revascularization SPTP was measured 24 h after PTA, at which point the lower limb microcirculation was unlikely to have recovered, meaning that the SPTP values could have been underestimated. Studies comparing the abilities of different agents and doses to induce maximal hyperemia are needed.

## 5. Conclusions

In this pilot study of isolated BTK lesions, lower peripheral FFR and SPTP values—both pre- and post-revascularization—were associated with a higher long-term risk of MALEs. Despite the limitation of a small sample size, this represents the first evidence supporting the long-term prognostic value of FFR in peripheral PTA. Larger studies are warranted to confirm these findings, clarify the role of FFR in BTK PTA, and establish a clinically relevant FFR cut-off value. FFR in BTK has the potential to measure the recruitment of microvascular perfusion improvement following revascularization, which may be less evident on plain angiography.

## Figures and Tables

**Table 1:** Clinical and procedural characteristics of the included patients.

Age, Years [IQR]	69 [62.5–74]
Male, N (%)	25 (62.5)
Body-mass index, kg/m^2^ [IQR]	28.2 [24.4–31.8]
Smoker, N (%)	22 (55.0)
Hypertension, N (%)	38 (95.0)
Dyslipidemia, N (%)	36 (90.0)
Renal impairment, N (%)	12 (30.0)
Diabetes mellitus, N (%)	25 (62.5)
Coronary artery disease, N (%)	12 (30.0)
Chronic obstructive pulmonary disease, N (%)	9 (22.5)
Atrial fibrillation, N (%)	7 (17.5)
Rutherford classification	
Category 3, N (%)	3 (7.5)
Category 4, N (%)	11 (27.5)
Category 5, N (%)	19 (47.5)
Category 6, N (%)	7 (17.5)
Artery revascularized	
Anterior tibial artery, N (%)	22 (55.0)
Posterior tibial artery, N (%)	12 (30.0)
Peroneal artery, N (%)	16 (40.0)
Ipsilateral presence of CTO in other BTK artery	16 (40.0)
Pre-revascularization FFR	0.645 [0.495–0.735]
Post-revascularization FFR	0.805 [0.65–0.875]
Absolute change in FFR	0.165 [0.075–0.25]
Percentage change in FFR, % [IQR]	24.6 [10.2–45.3]
Post-revascularization SPTP, mmHg	86 [60–96]

CTO, chronic total occlusion. BTK, below the knee. FFR, fractional flow reserve. IQR, interquartile range. SPTP, skin perfusion toe pressure.

**Table 2:** Results of multivariable survival analysis.

	MALE ^1^	Mortality ^2^
Pre-revascularization FFR (per 0.1-increment)	0.65 [0.46–0.93], *p* = 0.018	1.55 [0.96–2.51], *p* = 0.070
Post-revascularization FFR (per 0.1-increment)	0.40 [0.19–0.82], *p* = 0.012	1.07 [0.58–1.96], *p* = 0.833
Absolute difference in FFR (per 0.1-increment)	1.08 [0.51–2.28], *p* = 0.835	0.58 [0.17–2.02], *p* = 0.396
Percentage difference in FFR (per 10%-increment)	1.15 [0.89–1.48], *p* = 0.295	0.74 [0.43–1.29], *p* = 0.287
Post-revascularization toe pressure (per 10 mmHg-increment)	0.83 [0.71–0.98], *p* = 0.027	0.87 [0.64–1.19], *p* = 0.385

^1^ All estimates shown were adjusted sub-hazard ratios with the corresponding 95% confidence intervals. ^2^ All estimates shown were adjusted hazard ratios with the corresponding 95% confidence intervals.

## Data Availability

All underlying data are available on reasonable request to the corresponding author.
